# Alcohol Pattern Consumption Differently Affects the Efficiency of Macrophage Reverse Cholesterol Transport in Vivo

**DOI:** 10.3390/nu10121885

**Published:** 2018-12-03

**Authors:** Daniela Greco, Simone Battista, Laura Mele, Antonio Piemontese, Bianca Papotti, Stefania Cavazzini, Francesco Potì, Giulia Di Rocco, Andrea Poli, Franco Bernini, Ilaria Zanotti

**Affiliations:** 1 Biopharmanet-Tec Center, Università di Parma, Parco Area delle Scienze 27/A, 43124 Parma, Italy; daniela.greco@unipr.it; 2 Dipartimento di Scienze degli Alimenti e del Farmaco, Università di Parma, Parco Area delle Scienze 27/A, 43124 Parma, Italy; simone.battista91@gmail.com (S.B.); laura.mele3@gmail.com (L.M.); antonio.piemontese88@gmail.com (A.P.); bianca.papotti@gmail.com (B.P.); ilaria.zanotti@unipr.it (I.Z.); 3 Servizio di Medicina Preventiva dei Lavoratori, Università of Parma, Via Gramsci, 14, 43126 Parma, Italy; stefania.cavazzini@unipr.it; 4 Dipartimento di Medicina e Chirurgia-Unità di Neuroscienze, Università of Parma, via Volturno 39/F, 43125 Parma, Italy; francesco.poti@unipr.it; 5 Dipartimento di Scienze della Vita, Università degli Studi di Modena e Reggio Emilia, via Campi 103, 41125 Modena, Italy; giulia.dirocco@unimore.it; 6 Nutrition Foundation of Italy, Viale Tunisia 38, 20124 Milano, Italy; poli@nutrition-foundation.it

**Keywords:** alcohol, binge, reverse cholesterol transport, macrophages

## Abstract

It has been well established that moderate alcohol consumption inversely correlates with cardiovascular morbidity and mortality, whereas binge alcohol drinking increases cardiovascular disease risk. The aim of this study was to assess in vivo the impact of different drinking patterns on reverse cholesterol transport (RCT); the atheroprotective process leading to the removal of excess cholesterol from the body. RCT was measured with a standardized, radioisotope-based technique in three groups of atherosclerosis-prone apolipoprotein E knock out mice: Placebo group, receiving water, which would mimic the abstainers; moderate group, receiving 0.8 g/kg alcohol/day for 28 days, which would mimic a moderate intake; binge group, receiving 0.8 g/kg alcohol/day for 5 days/week, followed by the administration of 2.8 g/kg alcohol/day for 2 days/week, which would mimic a heavy intake in a short period. Mice in the binge drinking group displayed an increase in total cholesterol, high density lipoprotein cholesterol (HDL-c) and non-HDL-c (all *p* < 0.0001 vs. placebo), and a significantly reduced elimination of fecal cholesterol. The moderate consumption did not lead to any changes in circulating lipids, but slightly improved cholesterol mobilization along the RCT pathway. Overall, our data confirm the importance of considering not only the total amount, but also the different consumption patterns to define the impact of alcohol on cardiovascular risk.

## 1. Introduction

Alcohol consumption has been extensively studied in relation to its influence on many diseases, including cardiovascular disease (CVD), infection, cancer, and dementia [1,2]. Observational studies indicated that high doses of alcohol intake definitely result in organ damage [3,4], whereas moderate levels of alcohol consumption are likely to be safe and even beneficial for certain CVD, principally coronary heart disease [5]. Although the healthy potential of low-level alcohol consumption is still debated [6], a substantial body of prospective studies analyzed by Castelnuovo et al. in a recent meta-analysis revealed that light-to-moderate drinking (1–2 drinks per day for women, and 2–4 drinks per day for men) reduces cardiovascular events in a healthy population, with a 20–30% lower rate of CVD compared to abstainers [7]. On the contrary, binge drinking (more than 4 drinks consumed in a short period) has been associated with higher CVD risk compared to moderate consumption [8]. Based on this observation, the relationship between alcohol dosing and CVD mortality has been described as a J-shaped curve, with beneficial health effects associated with mild to moderate consumption, and increased CVD risk associated with higher doses of alcohol [9]. Strikingly, a very recent meta-analysis revealed the importance of persistent alcohol intake in cardiovascular risk assessment [10]. Possible mechanisms accounting for alcohol association to cardiovascular health have been suggested by epidemiological studies demonstrating that moderate alcohol consumption affects well-known risk factors and markers of CVD, such as inflammatory cytokines, adipokine levels, and fibrinogen [11]; in particular, modulators of the plasma lipoprotein profile. Moderate alcohol consumption has been shown to be associated with an increase in high density lipoproteins (HDL), and apolipoprotein A-I. It has also been associated with a decrease, despite usually small, in low density lipoproteins (LDL) [12]. In a recent study, Liu et al. showed the differential effect of alcohol drinking patterns on plasma lipids and atherosclerosis development in mice. In particular, they demonstrated that daily-moderate alcohol intake improves lipid profile, reducing LDL and increasing HDL, and decreases atherosclerotic plaque volume. Conversely, weekend binge alcohol intake, despite increasing HDL, is associated with increased plaque volume and macrophage accumulation in the neointima [13].

Interestingly, it has been demonstrated that the progression of atherosclerosis inversely correlates with the efficiency of the HDL-driven Reverse Cholesterol Transport (RCT) pathway, the process delivering excess cholesterol from peripheral tissues to the liver for the final elimination into the feces [14]. A pivotal player in RCT is the Scavenger Receptor Class B Type I (SR-BI). Originally described as a HDL receptor, this cell surface glycoprotein is highly expressed in the liver and steroidogenic tissues [15], where it promotes the bidirectional exchange of cellular cholesterol to HDL. Its role in RCT has been definitely demonstrated by Zhang et al., that revealed the positive regulation of the process in mice overexpressing SR-BI in the liver [16].

The aim of this study was to evaluate the effect of daily-moderate and weekend-binge patterns of alcohol consumption on the lipid profile and RCT from macrophages in vivo. For these purposes, athero-prone apolipoprotein E (apoE) knock out mice were administrated with different patterns of alcohol and the efficiency of whole macrophage-specific RCT was measured with a standardized, radioisotope-based technique [17]. In addition we investigated alcohol effects on the single steps of RCT in order to describe novel mechanisms accounting for the association of alcohol with cardiovascular health. We demonstrated that moderate alcohol consumption slightly impacts macrophage RCT, whereas binge consumption exerted several pro-atherosclerotic effects, including the increase of total- and non-HDL cholesterol and the impairment of cholesterol excretion from the body.

## 2. Materials and Methods

### 2.1. Animals

Thirty, 12 week-old male C57BL/6 (Envigo) and 29, 10 week old apo E^−/−^(B6.129P2-apoE ^tm1Unc/Crl^) mice (Charles River), were housed in a controlled environment and received a standard chow diet (Mucedola), water *ad libitum*. The alcohol feeding regimen was selected according to Liu et al. [13]. Apo E^−/−^ mice were divided into three groups: (1) Placebo (*n* = 9) receiving water for 28 days; (2) moderate (*n* = 10) receiving 0.8 g/kg alcohol/day for 28 days; and (3) binge (*n* = 10) receiving 0.8 g/kg alcohol/day for 5 days/week, followed by the administration of 2.8 g/kg alcohol/day for 2 days/week. In order to reach the same caloric intake from the treatment in all mice (0.49 kcal/mice/day), a supplementation of water and cornstarch was provided to the placebo and moderate groups. The former received 149.8 mg of cornstarch/mouse/day, and the latter 107 mg of cornstrach/mouse/day. Cornstarch and alcohol were dissolved in water and administrated by oral gavage.

The study was performed with the approval of the ethical Committee for Animal Experiments of the University of Parma.

### 2.2. Biometric and Clinical Analysis

Body weights were measured at baseline and on day 28 of treatment. Total cholesterol, HDL cholesterol (HDL-c) and triglycerides were quantified through the Aries system for Clinical Chemistry (ILab International, Milan, Italy), according to manufacturer’s instruction. Total cholesterol was evaluated by end-point analysis employing a colorimetric reaction (primary λ = 510 nm; secondary λ = 690 nm) producing a quinoneimine dye that is proportional to cholesterol in the sample. HDL-c was measured by end-point direct analysis (λ = 620 nm). Triglycerides were measured by end-point direct analysis employing a colorimetric reaction (primary λ = 510 nm; secondary λ = 690 nm) producing quinoneimine. Non-HDL-c was calculated as the difference between total and HDL cholesterol.

The activity of plasma alanine aminotransferase (ALT) was determined by a commercial kit (Sigma-Aldrich, St. Louis, MO, USA), according to manufacturer’s instruction. ALT activity was determined by a coupled enzyme assay, which results in a colorimetric product (570 nm) proportional to the pyruvate generated.

### 2.3. Macrophage RCT in Vivo

Macrophage RCT in vivo was evaluated through a standardised radiolabelled technique, in which macrophage-derived cholesterol is monitored along the RCT pathways [17,18]. Murine peritoneal macrophages (MPM) were obtained from C57BL/6 mice injected with 4 g/100 mL thioglycolate, 3 days before the sacrifice [19]. MPM were grown in suspension, radiolabeled with 5 μCi/μ [^3^H]-cholesterol, and enriched with 25 μg/mL of acetylated LDL (AcLDL) for 48 h. On day 26 of treatment with alcohol, 11.6 × 10^6^ cells/mouse containing 2 × 10^6^ counts per minute [cpm] were i.p. injected into apo E^−/−^ mice (1 mL/mouse). Blood and feces were collected 24 and 48 h after cell injection. After the sacrifice (day 28), blood was collected by cardiac puncture into plastic tubes containing EDTA K3 as anticoagulant. Plasma was obtained by low-speed centrifugation and counted in β-counter. Livers were excised after perfusion with a saline solution, weighed, and immediately frozen in liquid nitrogen. Hepatic and fecal sterols were extracted by the Bligh and Dyer method [20], and radioactivity was counted by liquid scintillation addition.

### 2.4. Hepatic Lipid Content

Liver samples were mechanically homogenized in H_2_O, extracted by the Bligh and Dyer method, and the lipophilic phase was evaporated under a stream of nitrogen. For cholesterol quantification, the Amplex Red^®^ Cholesterol Assay Kit (Molecular Probes, Eugene, OR, USA) was employed according to the manufacturer’s instructions. Hepatic triglycerides content was quantified through the Aries system for Clinical Chemistry (ILab International), as described above. Data were expressed as the ratio between lipid and protein cellular content, measured by a modified Lowry method [21].

### 2.5. Evaluation of Cholesterol Efflux Capacity (CEC)

The capacity of mouse plasma to promote cholesterol efflux, the CEC was measured through a standardized radiolabelled technique [22]. MPM were used as a cell model, in order to follow the conditions of in vivo RCT. MPM, withdrawn from thioglycolate-treated C57BL/6 mice as described above, were seeded into 24-wells plate, radiolabelled with 2 μCi/mL [^3^H]-cholesterol, and enriched with 25 μg/mL of acLDL for 24 h. Cells were equilibrated in 0.2% albumin-containing RPMI medium. Cholesterol efflux was promoted to 0.5% plasma of alcohol-treated apoE^−/−^ mice for 4 h and calculated as follows: Cpm [^3^H]-cholesterol in the medium / ([^3^H]-cholesterol in the medium + [^3^H]-cholesterol content in the monolayer) × 100.

### 2.6. Cholesterol Efflux Assay

MPM withdrawn from thioglycollate-treated C57BL/6 mice, as described above, were seeded into 24 wells-plates, radiolabelled with 2 μCi/mL [^3^H]-cholesterol and enriched with 25 μg/mL of acLDL for 24 h. Cells were equilibrated in 2% FCS-containing RPMI medium and treated with alcohol or acetaldehyde for 6 h. Cholesterol efflux was promoted to 0.5% control plasma from C57BL6 mouse for 4 h in the presence of alcohol or acetaldehyde. Cholesterol efflux was calculated as described in the previous paragraph.

### 2.7. RNA-Preparation, Reverse Transcription, and Quantitative Real-Time PCR (qPCR)

Total RNA was extracted using TRIzol^®^ Reagent (Life Technologies, Carlsbad, CA, USA), according to the manufacturer’s instructions. Five-hundred ng of RNA were retro-transcribed using a iScript™ cDNA Synthesis Kit (Bio-Rad Laboratories, Hercules, CA, USA). Real Time qPCR was executed in duplicate in a 96-well PCR plates (Bio-Rad Laboratories, Hercules, CA, U.S.A.) using SSo Fast Eva Green Supermix (Bio-Rad Laboratories, Hercules, CA, USA). mRNA levels were quantitatively detected using a CFX96™ Real-Time PCR Detection System (Bio-Rad Laboratories, Hercules, CA, USA). Relative gene expression was determined through the 2^−ΔΔCt^ method, using the *Hprt* gene as a housekeeping gene. Specific primers used to amplify the cDNA fragments were designed through Beacon Designer Software (Premier Biosoft International, Palo Alto, CA, USA). *Scavenger Receptor Class B type I* (*Scarb1*; sense: 5′ TTCTACTTGTCTGTCTACT 3′; antisense: 5′ ATTGAAGGTGATGTTGAC 3′); and *Hprt* (sense: 5′ ACCTGCTGGATTACATTA 3′; antisense: 5′ CTTCAACAATCAAGACATTC 3′).

### 2.8. Western Blot

Hepatic tissues were lysed in lysis buffer (0.5% Sodium Deoxycolate, 0,1% SDS, 1% Triton X-100, 20mM Tris Base, 150mM NaCl, and 5mM EDTA pH8), supplemented with a Protease Inhibitors Cocktail (1:100, Life Technologies, Carlsbad, CA, USA). After centrifugation, supernatants were isolated, sonicated, and protein concentrations were determined through BCA protein assay (Thermo Scientific, Rockford, IL, USA). Fourty µg of total protein was separated using 10% SDS-polyacrylamide gel electrophoresis, and then transferred to a PVDF Membrane. Nonspecific labeling was blocked by adding 5% non-fat-dried milk in TBST for 1 h. Membranes were incubated with primary rabbit anti mouse SR-BI antibody (Novus Biological, Littleton, CO, USA), overnight at 4 °C, and subsequently with HRP-conjugated donkey anti rabbit secondary antibody (GE Healthcare, Chicago, IL, USA). Bands were visualized by enhanced chemiluminescence (ECL, Thermo Scientific, Rockford, IL, USA). β-actin expression was used as control of loading after incubation with HRP-conjugated antibody recognizing β-actin (Sigma-Aldrich, St. Louis, MO, USA). Images were analyzed using ImageJ software (National Institute of Mental Health, Bethesda, MA, USA) by two independent blind observers.

### 2.9. Immunofluorescence Analysis of Liver Cryosections

Liver aliquots were fixed in formalin and embedded in Tissue-Tek OCT (Kaltek, Padua, Italy). Samples were processed for immunofluorescence microscopy by collecting 7 μm thickness sections using a cryostat. Sections were fixed with 10% Neutral Buffered Formalin in PBS, and permeabilized with 0.05% Triton X-100 (Sigma-Aldrich, St. Louis, MO, USA) in PBS solution. Nonspecific labeling was blocked by incubating samples with 1% BSA (Sigma-Aldrich, St. Louis, MO, USA), and 0.05% Triton X-100 in PBS solution. Liver cryosections were incubated with rabbit anti SR-BI (Novus Biological, Littleton, CO, USA). Sections were incubated for 1 h with goat anti rat AlexaFluor 488 conjugated antibody (Molecular Probes, Carlsbad, CA, USA), and mouse anti rabbit CruzFluor 594 conjugated antibody (Santa Cruz Biotechnology, Dallas, TX, USA). Slides were mounted with Fluoroshield^TM^ with DAPI histology mounting medium (Sigma-Aldrich, St. Louis, MO, USA), and coverslips were sealed. Images were acquired with a confocal microscope and SR-BI expression was determined using ImageJ software by quantifying the immunofluorescence intensity of liver cryosections (red signal) related to the total number of cells in each section.

### 2.10. Statistical Analysis

The statistical analyses were performed with Prism 6 software (GraphPad software, San Diego, CA, USA). The comparisons between placebo, moderate, and binge groups were analyzed by one-way ANOVA, followed by Tukey’s multiple comparison test between groups. A level of *p* < 0.05 was considered significant.

## 3. Results

### 3.1. Effect of Moderate and Binge Alcohol Consumption on the Plasma Lipoprotein Profile

Animals receiving moderate and binge alcohol displayed no significant changes in body weight from baseline to the end of treatment in comparison to control animals (Table 1).

At the end of the treatment period we measured liver weight and the activity of plasma ALT as markers of hepatocellular injury. No significant differences in plasma ALT levels or liver weight were observed among the groups. All together, these results suggest that animals displayed good health status throughout the experiment and that neither moderate nor binge intake of alcohol exerted adverse effects. As expected, alcohol consumption produced a slight increase in triglyceride hepatic content, more evident in the binge drinking group (Table 1).

Once verified that in our experimental conditions no signs of toxicity were evident, we evaluated the effect of drinking patterns on plasma lipid levels. We observed that binge, but not moderate alcohol consumption, significantly affected lipidemia. In detail, plasma total cholesterol significantly increased in mice receiving binge as compared to placebo (+45%), and moderate (+56%) groups. Mice receiving binge alcohol also showed higher levels of HDL-c compared to placebo (+98%), and moderate (+136%) groups. Interestingly, binge alcohol consumption also significantly increased non-HDL plasma cholesterol in comparison with placebo (+44%), and moderate (+54%) groups. Moderate and binge groups showed a tendency to higher triglycerides plasma levels compared to the placebo group, even though no statistically significant differences were reached (Table 1).

### 3.2. Effecst of Moderate and Binge Alcohol Consumption on the Reverse Cholesterol Transport in Vivo

**Figure 1:**
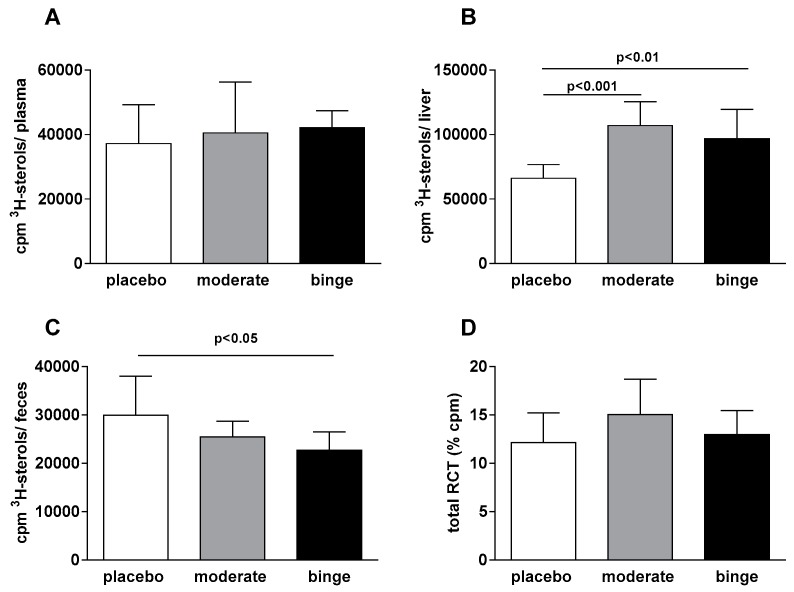
Effects of moderate and binge alcohol consumption on macrophage reverse cholesterol transport (RCT) in vivo. Apo E^−/−^ (B6.129P2-apoE^tm1Unc/Crl^) mice were treated with alcohol for 28 days as described in the Methods Section. On day 26 of treatment, animals were intraperitoneally injected with [^3^H]-cholesterol loaded murine peritoneal macrophages. After 48 h, mice were sacrificed and macrophage-derived [^3^H]-cholesterol distribution was quantified in plasma (**A**), liver (**B**), and feces (**C**). Total RCT was calculated as the sum of radioactivity detected in plasma, liver, and feces and expressed as percentage of [^3^H] dose injected (**D**). Results are expressed as mean ± SD (*n =* 9–10 mice per group) and were analyzed using one-way ANOVA followed by Tukey’s multiple comparison test among groups.

The impact of alcohol on the distribution of ^3^H-cholesterol along the RCT pathway was evaluated by measuring radioactivity content in plasma, liver, and feces. No significant differences among the three groups were evident in the amount of radioactivity of plasma 48 h after the injection of radiolabeled cholesterol (Figure 1A).

The amount of radioactivity in the liver was significantly higher in the moderate and binge groups compared to placebo (*p* < 0.001 and *p* < 0.01 respectively; Figure 1B), whereas the binge group excreted significantly less ^3^H-cholesterol at 48 h compared to the placebo (*p* < 0.05; Figure 1C). Overall, the removal of radioactivity from macrophages along the RCT pathway was higher, despite not statistically significant, in animals treated with a moderate dose of alcohol (Figure 1D), suggesting a slight promotion of the process.

### 3.3. Hepatic Cholesterol Content of Mice undergoing Moderate and Binge Alcohol Consumption

**Figure 2:**
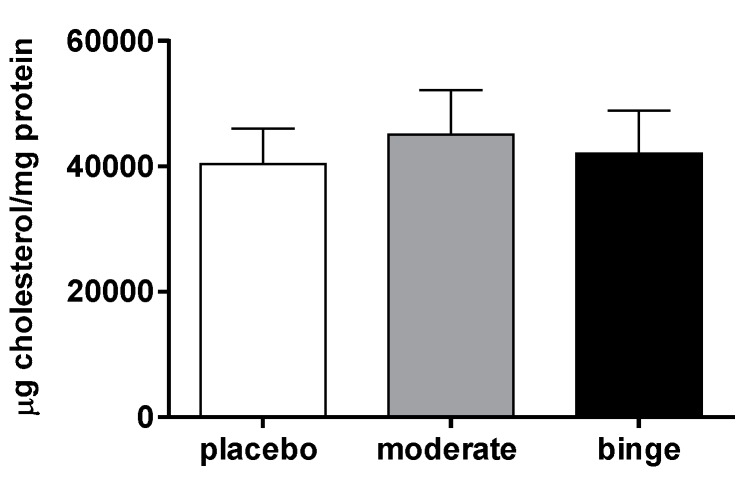
Hepatic cholesterol content in mice undergoing moderate and binge alcohol consumption. Apo E^−/−^(B6.129P2-apoE^tm1Unc/Crl^) mice were treated with alcohol for 28 days as described in the Methods Section. On day 28 of treatment, mice were sacrificed and the livers were collected after perfusion with a saline solution. Cholesterol was extracted according to Bligh and Dyer method, and successively quantified using a fluorimetric assay as described in the Methods Section. Data are presented as µg of cholesterol per mg of tissue proteins (mean ± SEM; *n =* 9–10 mice per group). Results were analyzed using one-way ANOVA followed by Tukey’s multiple comparison test among groups.

In order to investigate if the increased recovery of radioactive cholesterol associated with alcohol consumption was due to an accumulation of cholesterol in the liver, the total amount of hepatic cholesterol content was quantified. Moderate, as well as binge alcohol intake, did not influence the amount of total hepatic cholesterol (Figure 2), suggesting that the observed increase in hepatic cholesterol involved only the macrophage-derived cholesterol.

### 3.4. Effect of Moderate and Binge Alcohol Consumption on the First Step of RCT

**Figure 3:**
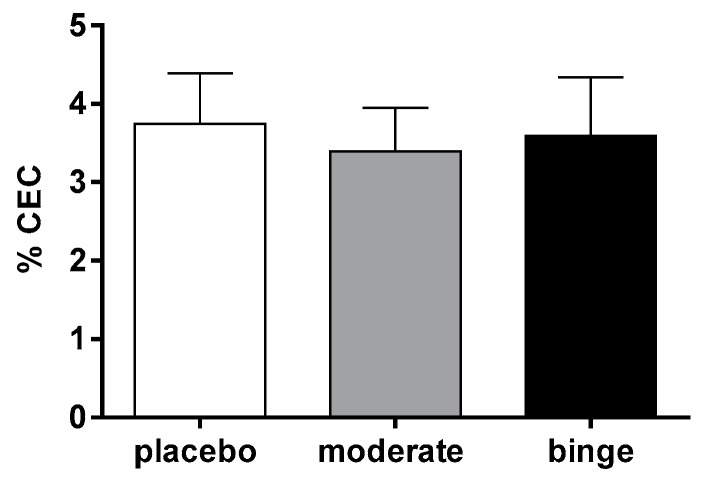
Effect of moderate and binge alcohol consumption on plasma cholesterol efflux capacity CEC. Murine peritoneal macrophages (MPM) were cholesterol enriched with 25 mg/mL acetylated LDL (AcLDL) and radiolabeled with [^3^H]-cholesterol 2 µCi/mL. After an equilibration period of 18 h, cells were exposed to 0.5% plasma from mice treated with placebo for 5 h, moderate or binge alcohol as described in the Methods Section. Efflux is expressed as cpm in medium/cpm of time zero × 100 (values are the mean of a triplicate). Data are expressed as mean ± SEM (n = 9–10 mice per group). Results were analyzed using one-way ANOVA followed by Tukey’s multiple comparison test among groups.

To assess whether the increased radioactivity in the liver was caused by a promotion of the first step of RCT, cholesterol efflux from macrophages, we followed two different approaches. In the former, we investigated the impact of different alcohol consumption patterns on the capacity of circulating HDL to promote cell cholesterol efflux. For this purpose, we incubated cholesterol-loaded macrophages with the plasma of mice consuming placebo, moderate, or binge alcohol. As shown in Figure 3, no difference in HDL function was observed, thus ruling out our hypothesis.

**Figure 4:**
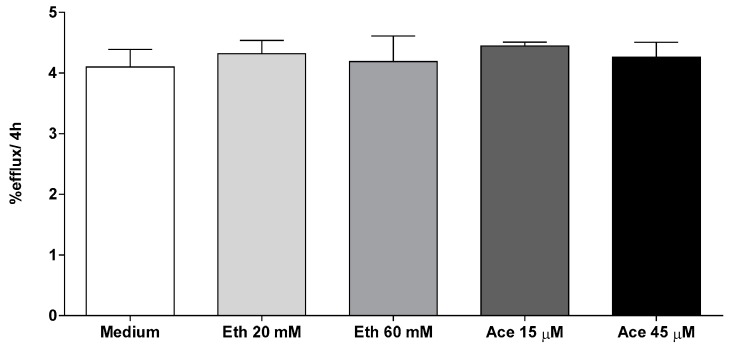
Effect of alcohol (Eth) and acetaldehyde (Ace) on cholesterol efflux from macrophages. MPM were cholesterol enriched with 25 µg/mL AcLDL and radiolabeled with [^3^H]-cholesterol 2 μCi/mL. Cells were equilibrated in 2% FCS-containing medium and treated with alcohol or acetaldehyde. Cholesterol efflux was promoted to 0.5% mouse plasma for 4 h in presence of acetaldehyde and alcohol as described in the Methods Section. Efflux is expressed as cpm in medium/cpm of time zero × 100 (values are mean of three triplicate). Data are expressed as mean ± SEM. Results were analyzed using one-way ANOVA followed by Tukey’s multiple comparison test among groups.

In the latter, we evaluated the direct effect of alcohol and its metabolite acetaldehyde on cholesterol efflux from macrophages. MPM were treated with two different concentrations of alcohol (20 mM and 60 mM) corresponding to the plasma concentration after a moderate and a heavy alcohol consumption in mice [13]. Similarly, acetaldehyde concentrations (15 μM and 45 μM) were selected in a range detected after alcohol intake in mice [23,24]. As shown in Figure 4, neither alcohol nor acetaldehyde affected cell capacity to eliminate cholesterol to extracellular acceptors. Therefore, also the involvement of increased macrophage efflux was not confirmed.

### 3.5. Effect of Moderate and Binge Alcohol Consumption on Hepatic Expression of SR-BI

**Figure 5:**
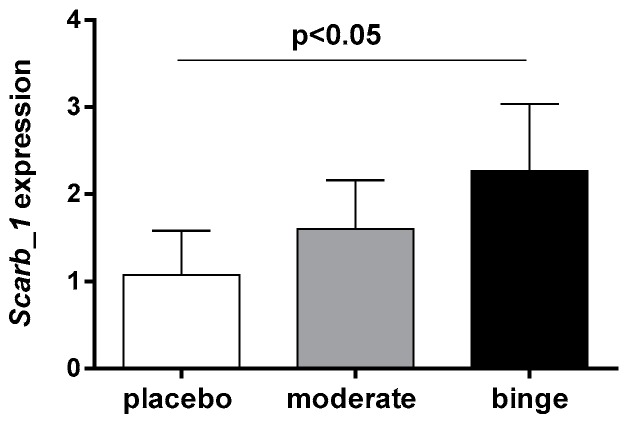
Effect of moderate and binge alcohol consumption on hepatic *Scarb1* gene expression. RNA was isolated from liver, as described in the Methods Section. *Scarb1* mRNA levels were determined by quantitative RT-PCR. *Hprt* was used as housekeeping gene for the normalization of *Scarb1* gene expression. Data are expressed as mean ± SD (*n =* 3–4 per group). Results were analyzed using one-way ANOVA followed by Tukey’s multiple comparison test among groups.

To investigate whether the increased radioactivity in the liver observed in moderate and binge groups was caused by a higher cholesterol uptake mediated by hepatic SR-BI, we quantified both gene and protein expression. As shown in Figure 5, *Scarb1* gene expression was higher in the binge group compared to placebo (*p* < 0.05), whereas no difference was observed between moderate and placebo groups.

**Figure 6:**
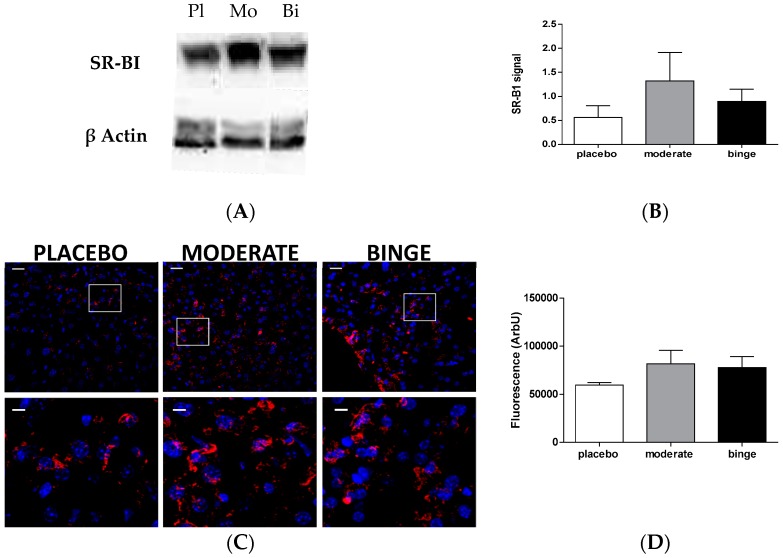
Effect of moderate and binge alcohol consumption on hepatic SR-BI protein expression. Western blotting analysis was performed on hepatic lysates, as described in the Methods Section. (**A**) Representative image of SR-BI expression in placebo (Pl), moderate (Mo), and binge (Bi) groups. (**B**) Signal quantification of SR-BI expression measured by ImageJ software. Signal quantification results were expressed as the ratio between SR-BI and the correspondent actin signal and data are expressed as mean ± SD (*n =* 3–4 per group). Results were analyzed using one-way ANOVA followed by Tukey’s multiple comparison test among groups. (**C**) Analysis of hepatic SR-BI expression (red signal) in placebo, moderate, and binge groups. Upper panels represent 20× magnification (thick = 25 µm); in lower panels, areas in the squares are enlarged (thick = 6 µm). (**D**) Results of red signal quantification were expressed as mean ± SD, after two independent analyses. Results were analyzed using one-way ANOVA followed by Tukey’s multiple comparison test among groups.

SR-BI protein expression in liver was evaluated through western blotting and confocal immunofluorescence microscopy. None of these techniques showed statistically significant differences in hepatic SR-BI protein expression, although a tendency to increased expression was observed both in moderate and binge groups compared to placebo (Figure 6). This evidence could, at least in part, explain the observed increase in macrophage-derived cholesterol in the liver.

## 4. Discussion

It is well accepted that, in addition to the amount consumed, the drinking pattern is a relevant factor influencing the impact of alcohol on CVD [8,10]. Whereas the intake of daily, moderate dose of alcohol is associated with lower incidence of cardiovascular mortality, the episodic intake of high amounts of alcohol concentrated over few hours causes a higher risk of acute events [25]. In the present paper we described the impact of different drinking patterns on the atheroprotective process of RCT in atherogenic prone ApoE knock out mice. Although these animals exhibited higher plasma cholesterol levels and spontaneous development of atherosclerotic lesions, the absence of systemic ApoE did not influence the efficiency of macrophages RCT [19]. The strain of animals was chosen in accordance with the work of Liu et al., who previously demonstrated that moderate alcohol consumption promotes the regression of atherosclerotic lesions, whereas binge intake caused their exacerbation [13]. The aim of our study was to investigate whether the different alcohol pattern could differently affect the specific removal of excess cholesterol from macrophages. Since our focus was to unravel alcohol effect on the efficiency of macrophage RCT rather than on the development of atheroma lesion, animals were fed a standard chow diet. Moreover, differently from the work by Liu et al., in our study the binge group received a moderate intake of alcohol for 5 days/week, overall mimicking a heavy consumption. It is important to underline that the selected treatment protocol did not negatively affect animal health, as indicated by the lack of differences in body and liver weights among groups, as well as the normal level of hepatic transaminase.

Mice receiving binge drinking displayed significant modifications of their plasma lipoprotein profile, with an increase in total cholesterol, HDL-c and non-HDL-c. These results were fully consistent with the effects of alcohol on lipoprotein pattern observed by Liu and colleagues [13]. Conversely, the moderate consumption did not produce significant changes in circulating lipids.

Under experimental conditions, the impact of alcohol consumption on the extent of macrophage RCT was modest. Interestingly, moderate and binge intake resulted in increased mobilization of cholesterol from macrophages to the liver. This result could be explained by the improvement of the first (cholesterol efflux from macrophages) or the second (hepatic cholesterol uptake) step of the process. To address the first question, we tested: (i) The capacity of alcohol and its primary metabolite acetaldehyde to directly affect cell cholesterol release; and (ii) the capacity of plasma from mice treated with placebo, moderate, or binge alcohol to promote cell cholesterol efflux. In fact, it is well known that cholesterol efflux from macrophages in vivo is influenced by (i) the capacity of cells to release cholesterol to extracellular acceptors, and (ii) the capacity of circulating lipoproteins to capture cholesterol from cells [22]. The first mechanism was supported by previous publications reporting alcohol capacity to affect cell cholesterol efflux. In a study performed in fibroblasts, cell exposition to concentrations of alcohol as found in plasma of heavy drinkers produced a reduction of cholesterol release to both apolipoprotein A-I and HDL [26]. Conversely, alcohol caused the increase of the expression and function of ATP Binding Cassette (ABC)A1 and ABCG1 transporters in astrocytes [27,28]. However, in our cultured, cholesterol-loaded macrophages, where ABCA1 and ABCG1 represent the key drivers of cholesterol efflux [29], neither alcohol nor acetaldehyde significantly affected cholesterol release. Thus, we could rule out the involvement of these transporters in the promotion of cholesterol mobilization from macrophages to the liver. Also, the second mechanism could be excluded, since plasmas from all groups displayed the same ability to take cholesterol from cholesterol-enriched macrophages.

To evaluate whether the increased appearance of radioactive cholesterol in the liver of alcohol-treated mice was related to an ameliorated hepatic uptake, we measured gene and protein expression of SR-BI, a well characterized HDL receptor [30]. Consistent with a recently published report [31], we observed that alcohol intake increased *SR-BI* gene expression compared to placebo. In particular, moderate consumption was related to the highest expression of SR-BI protein, an observation that was fully consistent with the trend of RCT. It is important to underline that this effect was not associated with a general raise of hepatic cholesterol mass, thus indicating that the specific delivery of macrophage-derived cholesterol was improved by alcohol.

Mice receiving binge alcohol presented similar amount of radioactive cholesterol in plasma and liver compared to the moderate drinking group, but showed a significant impairment of cholesterol elimination through the feces. This result indicated an additional deleterious effect of binge consumption, beyond the already reported alteration of lipoprotein profile. Moreover, it is worth to underline that the binge-driven raise of HDL cannot be interpreted as a beneficial effect, because these lipoproteins do not promote higher cholesterol efflux.

## 5. Conclusions

In conclusion, in this study moderate alcohol consumption in mice was associated with little impact on macrophage RCT, and no effect on lipoprotein profile. Interestingly, binge consumption, in the absence of hepatic toxic features, revealed several pro-atherosclerotic effects, including the increase of total and non-HDL cholesterol, the formation of dysfunctional HDL and the impairment of cholesterol excretion from the body. These data further confirm the importance of considering, at educational and clinical level, both the total amount and the different consumption patterns on the impact of alcohol on cardiovascular risk.

## Figures and Tables

**Table 1:** Effect of moderate or binge alcohol consumption on body weight, liver function, and plasma lipid profile.

	Placebo	Moderate	Binge
Body weight at baseline (g)	24.9 ± 1.5	24.3 ± 2.0	24.4 ± 2.3
Body weight on day 28 (g)	25.4 ± 1.8	25.6 ±1.3	24.5 ± 2.0
Liver weight (g)	1.10 ± 0.11	1.14 ± 0.22	1.25 ± 0.21
Hepatic triglycerides (mg/g liver)	9.5 ± 3.8	18.4 ± 4.3	27.5 ± 3.3 ^§§^
ALT (U/L)	2.83 ± 1.25	2.59 ± 1.22	3.18 ± 0.93
Total cholesterol (mg/dL)	328 ± 24	304 ± 39	478 ± 50 ^§§§§,^ ****
HDL-c (mg/dL)	11 ± 1	9 ± 1	22 ± 7 ^§§,^ ****
Triglycerides (mg/dL)	107 ± 25	151 ± 42	147 ± 26
Non-HDL-c (mg/dL)	316 ± 24	295 ± 39	455 ± 47 ^§§§§,^ ****

ALT = alanine aminotransferase; HDL = high density lipoproteins; **** *p* <0.0001 versus moderate; ^§§§§^ *p* < 0.0001 versus placebo; ^§§^ *p* < 0.01 vs. placebo.
